# Post-Imbrium Pb–Pb isochron ages for Apollo basaltic impact melt samples 14078 and 68415

**DOI:** 10.1098/rsos.231963

**Published:** 2024-05-15

**Authors:** Joshua F. Snape, Alexander A. Nemchin, Martin J. Whitehouse, Qiu-Li Li, Yu Liu, Nicholas E. Timms, Timmons Erickson, Gretchen K. Benedix

**Affiliations:** ^1^ Department of Earth and Environmental Sciences, University of Manchester, Manchester M13 9PL, UK; ^2^ Department of Applied Geology, Curtin University, Perth, Western Australia 6845, Australia; ^3^ Department of Geosciences, Swedish Museum of Natural History, 104 05 Stockholm, Sweden; ^4^ State Key Laboratory of Lithospheric Evolution, Institute of Geology and Geophysics, Chinese Academy of Sciences, Beijing 100029, People's Republic of China; ^5^ College of Earth and Planetary Sciences, University of Chinese Academy of Sciences, Beijing 100049, People's Republic of China; ^6^ Jacobs – JETS, NASA Johnson Space Center, Houston, TX 77058, USA

**Keywords:** Pb, Apollo, geochronology, isotope, impact

## Abstract

The Apollo 14 and 16 missions returned several samples commonly interpreted as crystalline impact melt, with ages of approximately 3800–3850 Ma. Previous work has suggested that these rocks formed in one or more pre-Imbrium basin forming impact(s). By contrast, recent ages determined for a range of lunar breccias provide compelling evidence that the Imbrium basin was formed at approximately 3920 Ma. Using an approach previously demonstrated in lunar basalts, Pb–Pb isochron ages are determined for two of these proposed impact melt samples (14078: 3848 ± 4 Ma; and 68415: 3834 ± 11 Ma). In the case of 14078, the least radiogenic Pb isotopic compositions measured here are interpreted as representing the initial Pb isotopic composition of the sample. This value indicates derivation from a source (or sources) with high ^238^U/^204^Pb ratios (approx. 2400), similar to those predicted for the Apollo 14 high-Al and very high-K basalts. It was not possible to determine an equivalent initial Pb isotopic composition for 68415, but Pb isotope evolution models indicate that the sample would have been derived from lithologies with lower ^238^U/^204^Pb source ratios (approx. 1000). In both cases, the samples are interpreted as having been formed by impacts local to the Apollo 14 and Apollo 16 landing sites.

## 1. Introduction

Constraining the impact bombardment chronology of the Moon remains one of the primary challenges in planetary science [1]. Early isotopic analyses of lunar samples collected during the Apollo missions provided the first evidence for a period of intense bombardment on the Moon at approximately 3.9 Ga [2,3], often referred to as the ‘Late Heavy Bombardment’ (LHB) [4]. Despite half a century of continued analysis of lunar impactite lithologies (i.e. shocked rocks, impact melt and breccias; see detailed discussion of definitions in [5]), there is continued debate over the timing, the intensity of impacts and the mechanism responsible for the LHB (e.g. [6–9]).

The formation of the Imbrium basin is considered to be the last basin-forming impact to have occurred during the LHB. As such, determining the absolute timing of the Imbrium basin forming event, and thereby a bracket for the end of the LHB, has been a major focus of lunar sample analyses (e.g. [10–16]). A detailed review by [17] demonstrated that the majority of Apollo 12, 14, 15, 16 and 17 impactite samples potentially linked to the Imbrium basin-forming event have similar ages, corresponding to a median value of 3922 ± 12 Ma. This age is significantly older than had been proposed in previous studies (e.g. 3750 Ma [10,11] or 3850 Ma [16]). While it remains impossible to unambiguously connect this age to a particular impact feature, the consistency across samples from the five separate landing sites, together with the wider geologic context of the samples and landing sites, led [17] to conclude that this age most likely represents the formation of the Imbrium basin.

The high flux of impactors associated with the LHB would have affected not only the Moon but also other planets within the inner Solar System, including the Earth. As such, confirming the intensity of a potential LHB and constraining the time at which it ceased is relevant not only to lunar geologic evolution but also the geologic evolution of the Earth. Indeed, it has previously been suggested that the timing of the LHB may be connected with the paucity of terrestrial rocks much older than approximately 3800 Ma [18] and that large impacts could have potentially even induced early terrestrial tectonics and mantle mixing [19]. Consequently, the difference between a proposed LHB ceasing at either 3750 Ma or 3920 Ma may provide the basis to support or reject such hypotheses.

Despite the consistency seen in the ages of many Apollo impactite samples, there are several outliers that do not fit with the 3922 ± 12 Ma age. For example, there are multiple samples from the Apollo 14 and 16 missions with ages that, when recalculated with updated decay constants, correspond to approximately 3850 Ma [17]. In the case of Apollo 14, these samples primarily comprise what have been commonly referred to as KREEP ‘basalts’ (KREEP being a geochemical signature defined by high concentrations of K, rare earth elements and P), despite the consensus that they represent crystalline impact melt [11,20–24]. The Apollo 16 samples in question include several crystalline impact melt samples (68415, 68416, 67559, 60635 and 65795 [10,16,23,25–27]) and the poikilitic impact melt breccia, 61569 [17,28]. Various interpretations of these Apollo 16 samples have been proposed previously, with suggestions that they may have formed before [10] or after [16] the Imbrium impact. Previous studies have defined distinct groups within the Apollo 16 impactite lithologies, with the majority of these crystalline impact melts (including 68415 and 68416) being classed as anorthositic noritic melt rocks (ANMR) [10,16,23] or comprising the group 3 categories in the schemes of [29] and [30].

In general, previous attempts to date impactite samples have largely focused on the ^40^Ar/^39^Ar technique (e.g. [11,26–28,31]), U–Pb dating of Ca-phosphate and zircon grains (e.g. [12,13,32]), and (less commonly) Rb–Sr isochron ages (e.g. [10,24,25,33]). Recent work with lunar basalts has demonstrated the potential of Pb–Pb isochron dating to provide precise crystallization ages [34–36]. As with all internal isochron dating approaches, the Pb–Pb method relies on the primary assumption that all of the analysed minerals in a particular sample are cogenetic. While crystalline igneous lithologies such as basalts may be the most obvious candidates to satisfy this criterion, the crystallization of melt generated by impact events could also be expected to produce samples suitable for the isochron dating approach. Indeed, the few Rb–Sr isochron ages pertinent to this discussion relate to crystalline impact melt [10,24,25,33,37].

In this study, Pb isotope compositions from mineral phases in two crystalline impact melt samples from the Apollo 14 (14078) and Apollo 16 (68415) missions were determined using secondary ion mass spectrometry (SIMS). These compositions have been used to constrain Pb–Pb isochrons to investigate when the rocks formed, as well as providing information about the Pb isotopic composition of the target lithologies that were melted. The ages determined from these isochrons are compared with those of related impactite samples from those missions and discussed in terms their relation to the impact bombardment history of the Moon.

## 2. Samples and methods

### 2.1. Sample descriptions

High-resolution backscattered electron (BSE) and transmitted light (plane-polarized and cross-polarized) images of the sections analysed in this study are available in the electronic supplementary material (figures S1–S3). Readers are also directed to the UK Virtual Microscope website (https://www.virtualmicroscope.org/), which hosts images of multiple Apollo 14 KREEP basalts (14073, 14074, 14077, 14276 and 14310) and two sections of 68415.

Sample 14078 is the largest (8.3 g) of several crystalline rocks (also including 14074, 14077, and 14079) collected from the bottom of a trench at Station G, approximately 250 m to the east of the lunar module. Together with several other samples (including 14073, 14310 and 14276), 14078 is referred to in the literature as a KREEP basalt [24,38]. This is despite early studies recognizing that the rocks likely represent samples of crystalline impact melt, based on a combination of mineralogy and petrography [20–22], as well as high siderophile element content [24].

**Figure 1.**
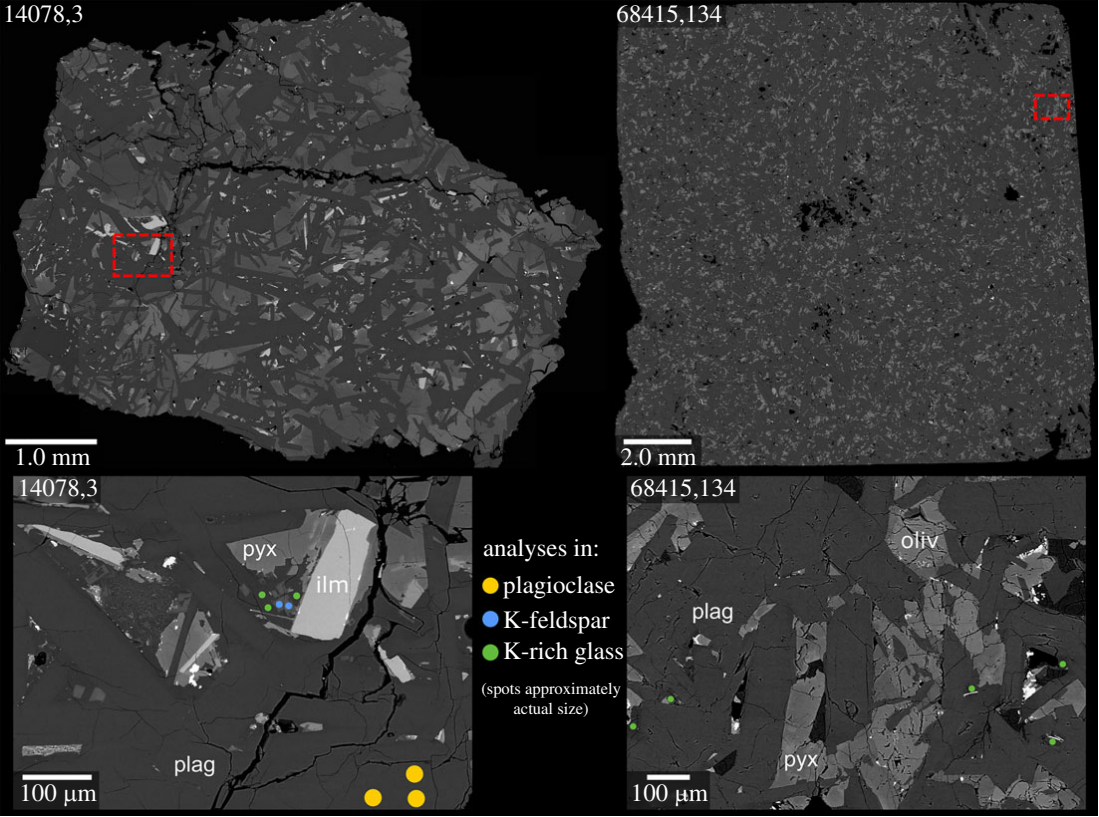
Backscattered electron images of the sections analysed in this study (14078,3 and 68415,146). The two bottom panels show more detail of the typical areas and phases analysed for Pb isotopic compositions.

The section (14078,3; figure 1) analysed in this study was also analysed by [24]. It has a subophitic texture, with plagioclase as the most common phase in the form of euhedral–subhedral laths (modal abundance = 62%; estimated from areal percentage of visible phases in the BSE images) up to 1 mm in length and typically 10–50 µm wide. The interstitial sites between the plagioclase grains are primarily occupied by anhedral grains of pyroxene (24%) and olivine (11%), typically 100–400 µm in size. Additionally, it contains grains of ilmenite (1%) and complex fine-grain (typically 1–10 µm) mesostasis assemblages comprising K-rich glass (1%), K-feldspar (<1%) and Ca-phosphate (<1%). There are also small (approx. 10 µm) anhedral grains of FeS scattered throughout the sample (<1%).

Samples 68415 and 68416 were sampled from a boulder at the edge of a 5 m crater, located in a ray of ejecta materials from South Ray Crater at the Apollo 16 landing site. As with the Apollo 14 KREEP basalts, the compositions and mineralogy of 68415 and 68416 were used as evidence for an impact melt origin, with the high liquidus temperature of the 68415 bulk composition, the evidence of a hybrid composition combining KREEP basalt and anorthosite lithologies, and experimental petrology studies highlighting the difficulty in producing similar melt compositions in the lunar interior [20,39]. Further support for an impact melt origin came from analyses of siderophile element concentrations in 68415, which were found to be higher than those found in mare basalts or anorthosites, and more similar to those found in breccias [40,41]. Early work on the samples also identified similarities between the Apollo 16 samples and the Apollo 14 KREEP basalts, particularly with regard to the high abundance of plagioclase in the rocks (approx. 80% in 68415 and 68416 [39,42–45]). The plagioclase in 68415 includes larger anhedral grains (calculated previously as 2–5% modally [39]), which have been interpreted as xenocrysts of relict unmelted material.

Descriptions of 68415 are provided in several previous studies [39,42,44–47], and the sections analysed in this study (68415,134 and, 146; figure 1) are consistent with these. Plagioclase is the most abundant phase (81%), with a number of larger anhedral grains (comprising approximately 5% of the plagioclase in the sections; i.e. approximately 3% of the total sample) up to 3.0 mm in length and 0.3 mm wide, within a fretwork of randomly oriented anhedral to euhedral laths typically around 100–300 µm long and 50–100 µm wide. Pyroxene (11%) and olivine (4%) are present in the interstitial sites between the plagioclase, with grains rarely larger than 100 µm in size. Other phases in these interstitial sites include K-rich glass (3%), silica (1%), ilmenite (<1%) and FeS (<1%).

### 2.2. Analytical methods

Following the SEM documentation of the samples and prior to the SIMS analyses, the samples were cleaned with a fine (1 µm) diamond paste and ethanol to remove the carbon coating before adding a 30 nm gold coating. The Pb isotopic compositions (complete dataset presented in electronic supplementary material, table S1) of the phases were determined over three analytical sessions using a methodology similar to that outlined in previous studies (e.g. [34,48]). The first two sessions were conducted using the CAMECA IMS 1280 ion microprobe at the NordSIMS facility, Swedish Museum of Natural History, Stockholm. The final session was conducted using the CAMECA IMS 1280HR ion microprobe at the Institute of Geology and Geophysics, Chinese Academy of Sciences (IGGCAS), Beijing. Apertures in the primary column were used to generate a slightly elliptical O_2_^−^ sample probe with dimensions appropriate to the target. The smaller accessory phases (including K-feldspar and phosphates) were analysed using a 10 µm spot (beam current 3–6 nA), whereas larger grains (including plagioclase and K-rich glass) were analysed with a 20 µm spot (beam current 16–20 nA). Prior to each measurement, an area of 15–25 µm around the spot location was rastered for at least 120 s to remove the gold coating and minimize possible surface contamination. The instrument was operated in high-transmission mode, corresponding to a transfer magnification of 160×. In this mode, the field aperture size was chosen to limit the field of view on the sample surface (i.e. the area from which ions will be admitted to the mass spectrometer) to be slightly larger than the spot but smaller than the rastered area, further minimizing the possibility of measuring surface contamination. The mass spectrometer was operated with a nominal mass resolution of 4900 (*M*/Δ*M*) (4860 for the NordSIMS sessions and 5000 for the IGGCAS session), sufficient to resolve Pb from known molecular interferences. A nuclear magnetic resonance (NMR) field sensor regulated the stability of the magnetic field to high precision. The Pb isotopes were measured simultaneously in multi-collector mode using four low-noise (less than 0.006 counts per second) ion counting electron multipliers (Hamamatsu 416) with electronically gated deadtimes of 65 ns. Background counts for each channel were measured at regular intervals during each session by using deflector and aperture settings that effectively blank both primary and any residual secondary beams. The average background values for each session are reported in electronic supplementary material, table S2. Individual analyses were filtered out of the final dataset if the count rates for any of the masses were less than 3× the average background count rates during that session.

Analyses of the USGS basaltic glass reference material BCR-2G were used to generate a correction factor to account for mass fractionation and detector relative gain calibration in the unknown analyses. This correction procedure involved dividing each of the ‘accepted’ isotope ratios for BCR-2G (determined independently using TIMS analyses [49]) by the corresponding average of each ratio obtained from all standards in a given session in order to obtain a ratio-specific correction factor that incorporates both mass bias (a few parts per thousand at Pb mass [50]) and inter-detector (a few percent) gain (electronic supplementary material, table S3). Isotope ratios of unknown samples were then corrected by multiplying by these factors. Within uncertainty limits, no systematic drift was observed in the BCR-2G measurements during a given analytical session, with the 1*σ* standard deviations from the average session values all being less than 1% (the complete set of average session values and associated standard deviations are reported in electronic supplementary material, table S3).

The uncertainties stated for each ratio in the individual sample measurements (electronic supplementary material, table S1) are derived from the internal run error propagated together with the standard deviations of the BCR-2G analyses for the relevant analytical session and the uncertainty given for the published BCR-2G values [49]. The errors stated for the Pb–Pb isochron dates and initial Pb isotope composition of 14078 in the following Results and Discussion sections are quoted at the 95% confidence level.

### 2.3. Data processing

**Figure 2.**
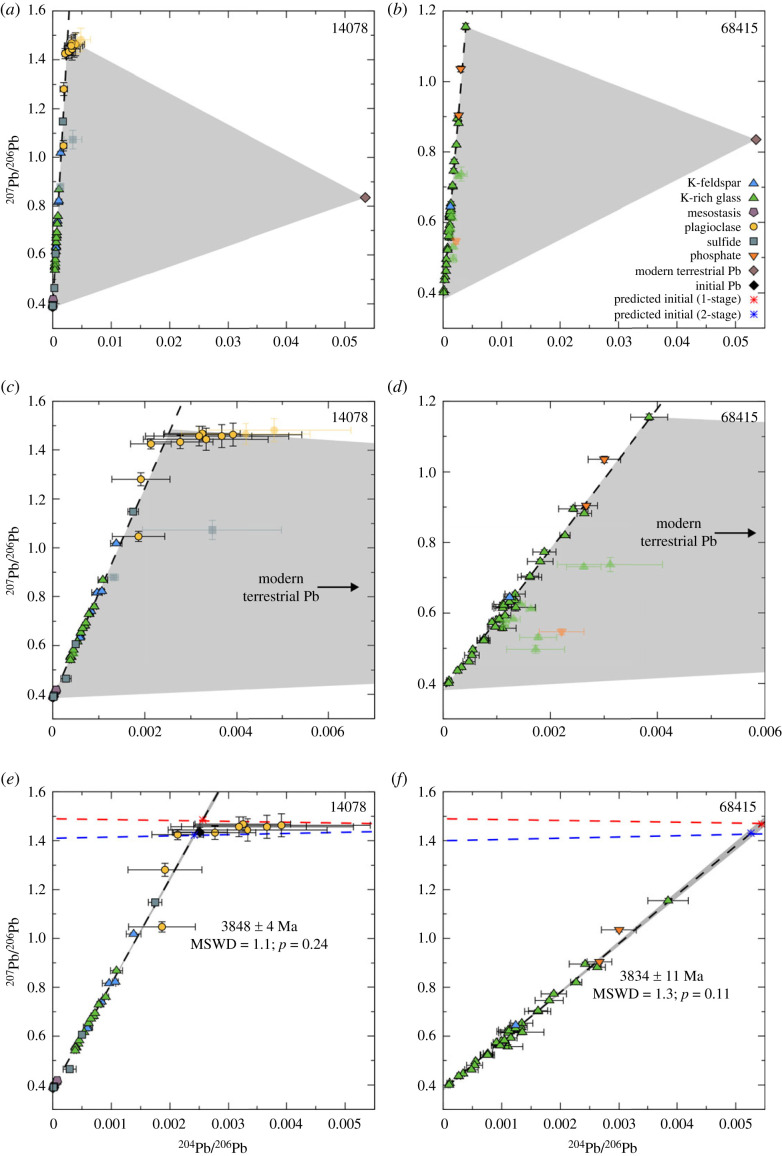
Plots of ^207^Pb/^206^Pb versus ^204^Pb/^206^Pb for the data collected from 14078 and 68415. The assumed mixing relationship between the most radiogenic Pb, initial Pb and modern terrestrial Pb is illustrated by the grey triangles in (*a*,*b*). The vast majority of analyses fall close to the leftmost edges of these triangles, as can be seen when focusing on this region of each plot (*c*,*d*). Pb–Pb isochrons for (*e*) 14078 and (*f*) 68415 are generated by filtering the few points in each dataset that do not lie within uncertainty of the leftmost edge. Error bars represent 2*σ* uncertainties.

The data were processed using in-house SIMS data reduction spreadsheets and IsoplotR [51], using the same approach as that outlined in [34]. Briefly, the Pb isotopic compositions measured in each sample (electronic supplementary material, table S1) are interpreted as representing a mixture between three main components: (1) initial Pb present in the impact-melted target material when it crystallized, (2) radiogenic Pb formed by the decay of U in the rocks after crystallization, and (3) terrestrial contamination. These endmember components define a triangular array of points on a plot of ^207^Pb/^206^Pb versus ^204^Pb/^206^Pb (examples of this are shown in figure 2*a*,*b* using the 14078 and 68415 datasets). The values with the highest ^207^Pb/^206^Pb ratios, at the top of the triangular array, will provide an estimate of the lowest possible value for the initial Pb composition of the sample. The radiogenic Pb component will be located where ^204^Pb/^206^Pb = 0. Finally, given the radiogenic Pb isotopic compositions associated with the Moon relative to those found on Earth, the terrestrial contamination endmember will have highest ^204^Pb/^206^Pb ratios. The reliability of this approach has now been demonstrated in multiple previous studies [34–36,52], notably including the analysis of samples that have been previously dated using multiple different chronometers. The assumed three-component mixing is also consistent the results in this study, particularly taking the example of 14078, where the broadest range of phases were analysed provided the widest range of Pb isotope compositions. Given that the Moon is generally depleted in Pb, the highest concentrations of Pb in lunar rocks tend to be associated with the *in situ* decay of U after the sample has formed, while initial Pb is present in much lower concentrations. This is consistent with the 14078 analyses, where the highest Pb counts are observed in the most radiogenic analyses, while those values closer to the initial compositions have significantly lower counts and tend to be more susceptible to the effects of terrestrial contamination.

Based on this three-component mixing assumption, the bounding edge on the left side of the triangle, between the initial and radiogenic lunar Pb compositions, forms an isochron. This isochron was determined by fitting a straight line through the data [53] and iteratively filtering the individual data points to exclude those with high weighted residual values (greater than 2) for each regression, so as to yield the steepest statistically significant weighted regression (i.e. MSWD less than 2; probability of fit, *p* > 0.1 [54]). The age of each sample is then determined by the *y*-intercept of the isochron (i.e. the radiogenic ^207^Pb/^206^Pb ratio), using the decay constants *λ*^238^U = 1.55125 × 10^−10^ yr^−1^ and *λ*^235^U = 9.8485 × 10^−10^ yr^−1^ [55,56], and assuming a present-day ^238^U/^235^U ratio of 137.818 [57].

## 3. Results

The phases targeted in 14078,3 for Pb isotopic analyses included plagioclase, Fe-sulfide, K-rich glass, K-feldspar and several fine-grained (less than 1 µm grainsize) mesostasis assemblages. A total of 51 analyses were made, of which 6 were filtered out due to evidence of terrestrial contamination (figure 2*c*), leaving a 45-point Pb–Pb isochron that defines a date of 3848 ± 4 Ma (MSWD = 1.1; *p* = 0.24; figure 2*e*). A cluster of 5 plagioclase analyses at the top of the isochron ratios define a single population with an X-Y weighted average value of ^204^Pb/^206^Pb = 0.0025 ± 0.0005 and ^207^Pb/^206^Pb = 1.44 ± 0.02 (^206^Pb/^204^Pb = 400 ± 80; ^207^Pb/^204^Pb = 576 ± 116; MSWD = 1.5; *p* = 0.16; figure 2*e*). A weighted average of the same points also yields ^208^Pb/^206^Pb = 0.8439 ± 0.0091 (^208^Pb/^204^Pb = 337 ± 68; MSWD = 0.86; *p* = 0.48). The most radiogenic compositions in the sample, defined as those with the lowest ^207^Pb/^206^Pb and ^204^Pb/^206^Pb ratios, were obtained from the mesostasis assemblages.

Analyses of 68415,134 and 68415,146 sections focused primarily on K-rich glass, although analyses were also made of K-feldspar and Ca-phosphate. Of the 47 analyses that were significantly above the background detection limits, 10 were identified as showing signs of terrestrial contamination (figure 2*d*). The remaining 37 analyses form an isochron corresponding to a date of 3834 ± 11 Ma (MSWD = 1.3; *p* = 0.11; figure 2*f*). Both the top and bottom ends of the isochron are defined by analyses of K-rich glass, with the highest ^207^Pb/^206^Pb and ^204^Pb/^206^Pb ratios obtained from a single analysis (^204^Pb/^206^Pb = 0.0032 ± 0.0003; ^207^Pb/^206^Pb = 1.15 ± 0.01; ^208^Pb/^206^Pb = 0.963 ± 0.008; ^206^Pb/^204^Pb = 260 ± 12; ^207^Pb/^204^Pb = 300 ± 13; ^208^Pb/^204^Pb = 250 ± 11). Two Ca-phosphate and one K-feldspar are included in the filtered dataset comprising the isochron. Notably, the Ca-phosphate analyses have less radiogenic Pb isotope compositions (i.e. plotting towards the top of the isochron) than would typically be expected from such phases. Equally, the K-feldspar analysis is more radiogenic (i.e. lower down the isochron) than would typically be expected. In both cases, this is most likely due to the small nature of the targeted phases meaning that an adjacent phase was included in the analysed area of the sample.

Given the crystalline natures of both rocks and the lack of petrographic evidence for subsequent alteration, together with the statistically robust isochrons, these dates are interpreted as most likely representing impact melt crystallization ages.

## 4. Discussion

### 4.1. Comparison with previous age determinations

In order to compare the new Pb–Pb isochron ages with earlier studies, the previous age determinations were recalculated using modern decay constants. For ^40^Ar/^39^Ar, the values of [58,59] were used (*λ*^40^K_tot_ = 5.5305 × 10^−10^ yr^−1^). The values of [60] were used for recalculating the Rb–Sr ages (*λ*^87^Rb = 1.3972 × 10^−11^ yr^−1^). A single Sm–Nd study was also relevant to this discussion (the 14150,7,3 analyses by [61]), which already used the commonly accepted value of *λ*^147^Sm = 6.54 × 10^−12^ yr^−1^ [62].

#### 4.1.1. 14078 and other Apollo 14 crystalline melt rocks

**Figure 3.**
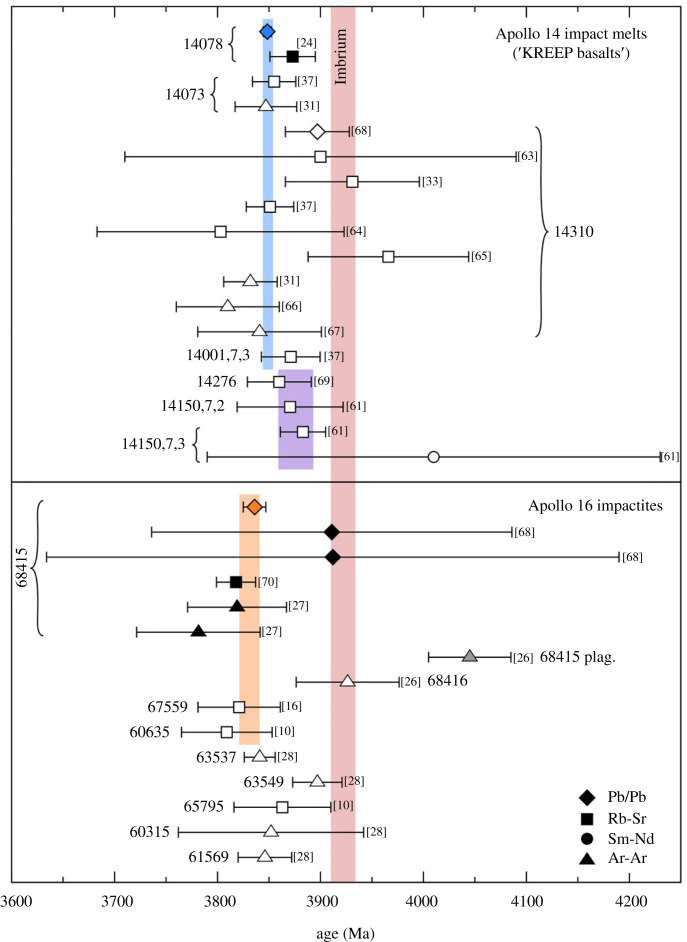
A comparison of the new Pb–Pb isochron ages for 14078 and 68415 with previously determined ages for the same samples and other associated samples from the same landing sites. The 14078 age is compared with the ages of the other Apollo 14 KREEP basalts. The blue and purple coloured bars indicate the weighted average ages for the two groups of Apollo 14 KREEP basalts identified by [61]. The 68415 age is compared with the other ANMR/group 3 Apollo 16 samples, as well as the similarly aged impact melts 65795 and 60315, and the impact melt breccia 61569. The orange coloured bar indicates the weighted average age for the group 3 samples identified by [30] as being compositionally similar. The light red coloured bar indicates the age of the Imbrium basin, as calculated by [17].

There is only one previous dating study of 14078 [24], which produced a well-defined Rb–Sr isochron equating to an age of 3873 ± 22 Ma, but multiple previous studies have determined ages for several of the other Apollo 14 crystalline impact melt samples collected at the same location (i.e. the other Apollo 14 ‘KREEP basalts’). The 14310 sample has been the subject of the most dating studies, including five Rb–Sr studies [33,37,63–65], three ^40^Ar/^39^Ar studies [31,66,67], and a U–Pb study [68]. Out of these nine studies, six are in good agreement with the 14078 Pb–Pb isochron age determined here (figure 3). Two of the Rb–Sr studies [33,65] yielded older ages (3931 ± 65 Ma and 3966 ± 78 Ma, respectively) than that from the 14078 Pb–Pb isochron, but in both cases are determined from Rb–Sr isochrons with very low probabilities of fit (*p* = 0.003 and 0.001; MSWD = 3.1 and 4.6). The previous U–Th–Pb SIMS analyses [68] of accessory phases in a range of samples, including an ‘Y-REE’ phase in 14310, yielded a ^207^Pb/^206^Pb ratio of 0.3968, equating to an age of 3896 ± 31 Ma. It should be noted that this age is calculated assuming that the measured ^207^Pb/^206^Pb ratio represents pure radiogenic Pb resulting from the *in situ* decay of U after the sample crystallized, and has not been corrected for the presence of any initial Pb. Furthermore, the lack of a ^204^Pb measurement precludes performing such a correction. This is particularly important given the very radiogenic (even by lunar standards) nature of the Pb isotope compositions in these Apollo 14 samples and their association with the geochemical KREEP signature. As such, an uncorrected ^207^Pb/^206^Pb ratio will very likely result in an artificially older age, providing an explanation for the offset between the 14310 ^207^Pb/^206^Pb age and the 14078 Pb–Pb isochron age determined in the present study.

In addition to 14310, Rb–Sr and ^40^Ar/^39^Ar analyses of 14073, 14276 and 14001,7,3 [31,37,69] also yielded ages that are in good agreement with the 14078 Pb–Pb isochron (figure 3). In [61] fragments were analysed from the soil sample 14150, which was also collected from the Station G trench. The Rb–Sr isochron obtained for one of these fragments (14150,7,2) yielded an age of 3843 ± 35 Ma, although [61] note a potentially suspect plagioclase datapoint which, if omitted, results in a slightly older isochron age of 3871 ± 52 Ma. The second fragment (14150,7,3) yielded a Rb–Sr isochron age (3883 ± 22 Ma), as well as a less precise Sm–Nd age (4010 ± 220 Ma). Finally, ^40^Ar/^39^Ar ages were determined for two other crystalline impact melt samples (14074 and 14079) collected from the Station G trench [11], but there is insufficient information to recalculate these with modern decay constants.

Based on variations in Sr isotope compositions (see discussion below; §4.2), it was previously concluded that the Apollo 14 KREEP basalts were divided into at least two distinct groups derived from separate events [61]. Excluding the 14310 ^207^Pb/^206^Pb age and the Rb–Sr isochrons with low probabilities of fit, a weighted average age for the group including 14078 (14073, 14078, 14310 and 14001,7,3) is 3849 ± 5 Ma (MSWD = 1.3; *p* = 0.24; figure 3). Meanwhile, a slightly older weighted average age of 3876 ± 17 Ma (MSWD = 1; *p* = 0.39) is calculated for the remaining Apollo 14 KREEP basalts (14276; 14150,7,2; and 14150,7,3).

#### 4.1.2. 68415/68416 and other Apollo 16 crystalline melt rocks

There have been several dating studies focused on 68415 and 68416, with the first being the Rb–Sr analyses of [25], yielding an internal isochron equating to an age of 3818 ± 19 Ma for 68415. Subsequent analyses of 68416 by the same authors were added to the 68415 dataset, resulting in a combined Rb–Sr isochron age of 3821 ± 17 Ma [70]. A further Rb–Sr isochron for 68416 was presented by [71], indicating a similar age of 3853 ± 30 Ma. Two ^40^Ar/^39^Ar studies of the rocks yielded ages for 68415 of 3819 ± 48 Ma [27] and 3782 ± 60 Ma [26] based on whole rock samples. Plagioclase separates from 68415 were also analysed by [27], resulting in an older age (4045 ± 40 Ma), while [26] determined an older age (3926 ± 50 Ma) for 68416. These older ages were interpreted as potentially resulting from the incorporation of older relict material in the impact melt [26]. A separate, slightly younger ^40^Ar/^39^Ar age (3770 ± 40 Ma) was determined by [67], but it is worth noting that this is defined by a high-temperature plateau comprising a relatively small fraction (approx. 25%) of the released ^39^Ar. Furthermore, it was not possible to obtain revised ages for the monitor used in that study. In addition to the Rb–Sr and ^40^Ar/^39^Ar studies, [68] presented ^207^Pb/^206^Pb ratios for a Ca-phosphate and a Yr–Zr phase in 68415, equating to ages of 3911 ± 175 Ma and 3911 ± 278 Ma, respectively. As with their analyses of 14310, it should be noted that these ^207^Pb/^206^Pb ratios have not been corrected for the presence of initial lunar Pb. In this case, the relative lack of KREEP-rich material in the Apollo 16 rocks, when compared with those from Apollo 14, means that the presence of any uncorrected initial Pb would have a less extreme effect on the ^207^Pb/^206^Pb age. Furthermore, any such correction would likely be insignificant, relative to uncertainties quoted for the 68415 ^207^Pb/^206^Pb ratios, which are much larger than those for 14310 [68]. Taken together, the majority of these ages are in good agreement with the new Pb–Pb isochron for 68415 (figure 3).

It is also noteworthy that several other ANMR/group 3 [10,16,30] Apollo 16 impactite samples have been found to have similar ages to 68415 and 68416. Analyses of 67559, a similarly plagioclase-rich subophitic impact melt rock, yielded an internal Rb–Sr isochron reflecting age of 3821 ± 40 Ma [16]. Deutsch & Stöffler [10] performed Rb–Sr analyses for the crystalline impact melt sample 60635, with the authors identifying two regressions, equating to 3809 ± 44 Ma (excluding analyses of clinopyroxene) and 3932 ± 16 Ma (excluding a mesostasis analysis). Similar to the older 68415 plagioclase and 68416 dates, the 60635 dates were interpreted as representing the presence of older impact melt rock material, which was subsequently incorporated in the melt generated by a second impact [10]. Excluding the older ^40^Ar/^39^Ar and ^207^Pb/^206^Pb ages for 68415 and 68416, a weighted average age of all the ANMR/group 3 samples is 3831 ± 10 Ma (MSWD 1.2; *p* = 0.28).

The remaining group 3 samples are 63537, 63549 and 65055, which [30] noted were compositionally distinct from the other group 3 samples, having slightly lower REE concentrations and higher Ir/Au ratios. When recalculated for updated decay constants and monitor ages, ^40^Ar/^39^Ar plateaus for 63537 and 63549 [28] indicate ages of 3841 ± 15 Ma and 3897 ± 24 Ma, respectively, suggesting that at least 63549 was indeed formed during a separate impact event from the other group 3 samples. For 65055, there is only a single old ^40^Ar/^39^Ar age determination [72], with insufficient information to recalculate for updated decay constants.

Beyond the ANMR/group 3 samples, several other Apollo 16 impactites have potentially similar ages to 68415. For example, [10] obtained a Rb–Sr isochron for 65795, indicating an age of 3863 ± 47 Ma. Despite the similarity in age to the ANMR/group 3 samples, compositional distinctions, including lower Sc and REE concentrations, suggest 65795 derived from a separate melt sheet [10,30]. Finally, ^40^Ar/^39^Ar ages for the poikilitic melt breccia 61569 and the impact melt 60315 indicate that these rocks formed at 3846 ± 26 Ma and 3852 ± 90 Ma, respectively [17,28]. Sample 61569 was not grouped by [30], but it has higher trace element concentrations than the group 3 samples, and the similarity in age may be coincidental. In the case of 60315, which was classified as group 1, [73] determined Pb isotope compositions that form an isochron equating to approximately 3935 Ma. The authors originally determined an uncertainty of ±4 Ma for this value, although this is difficult to verify, due to a lack of explicitly stated measurement uncertainties. The combined ^40^Ar/^39^Ar and Pb isotope data suggest that 60315 may be older than 68415, and potentially within uncertainty of the Imbrium basin formation, and consistent with the majority of group 1 and 2 samples.

### 4.2. Isotopic compositions of the samples and implications for impact target lithologies

#### 4.2.1. 14078

**Figure 4.**
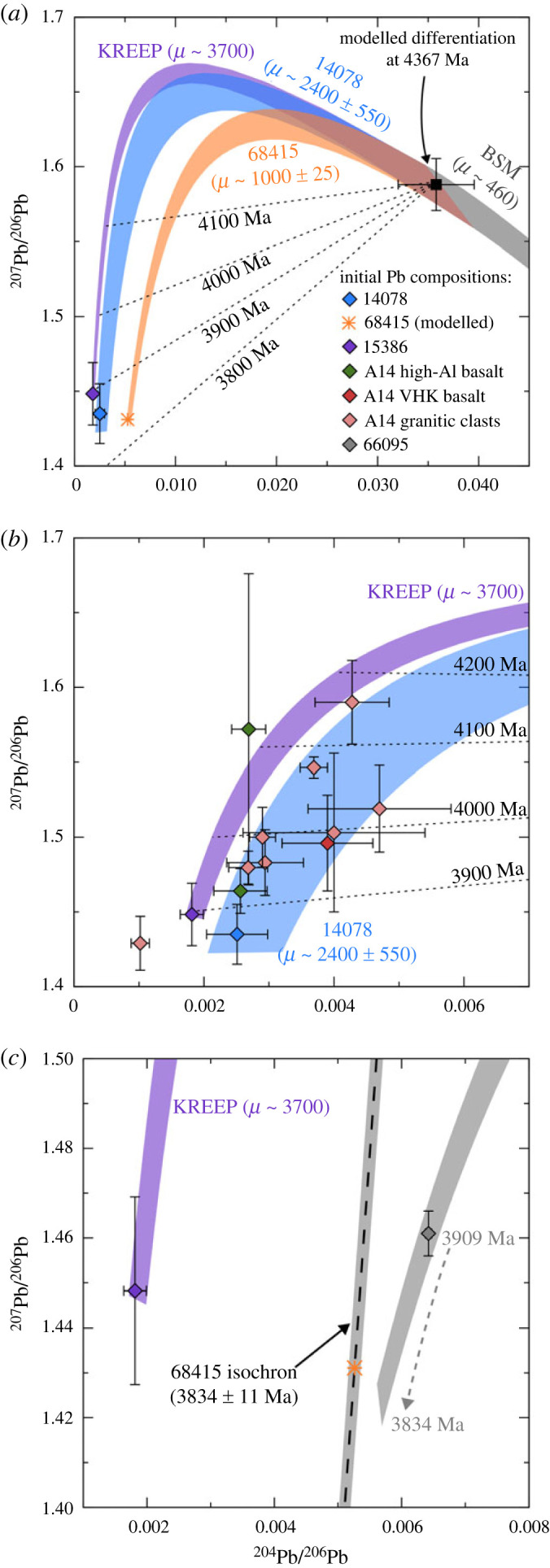
(*a*) Pb isotope growth curves based for the initial Pb compositions of 14078 (measured) and 68415 (modelled), using the multiple stage model of [34]. In this model, the bulk silicate Moon evolves with a µ-value of approximately 460 until a differentiation event at 4367 Ma, likely signifying the end of magma ocean crystallization. Also plotted is a growth curve and initial Pb composition for the Apollo 15 KREEP basalt 15386. (*b*) Initial Pb composition of 14078 compared with Apollo 14 high-Al basalts [34,52], VHK basalts and granitic clasts [76,77]. The majority of these compositions can be explained by a similar source µ-value of approximately 2400. (*c*) Modelled initial Pb composition of 68415 compared with the measured composition of impact melt breccia 66095 [78]. The composition of 66095 appears to indicate a slightly lower contribution of KREEP than is present in 68415, given that a growth curve extrapolated from the age of 66095 (3909 Ma) until that of 68415 (3834 Ma) fails to intercept the 68415 axis. All error bars and growth curve fields indicate 2*σ* uncertainties.

**Figure 5.**
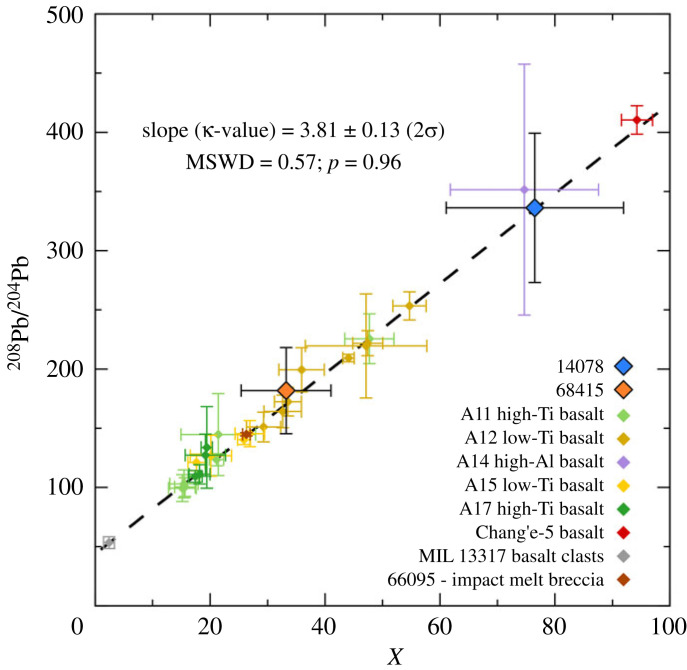
Plot of the best estimates for the initial ^208^Pb/^204^Pb compositions for 14078 and 68415 plotted against $X=(e^{\lambda_{232}t_{1}}-e^{\lambda_{232}t_{i}}/e^{\lambda_{238}t_{1}}-e^{\lambda_{238}t_{i}})\times ({\;}^{206}\mathrm{Pb}{/}^{204}{\mathrm{Pb}}_{i}\;-{\;}^{206}\mathrm{Pb}{/}^{204}{\mathrm{Pb}}_{1})$, such that the slope of the trend is equal to the κ-value of the system, given that: ${\;}^{208}\mathrm{Pb}{/}^{204}{\mathrm{Pb}}_{i}=\kappa (e^{\lambda_{232}t_{1}}-e^{\lambda_{232}t_{i}}/e^{\lambda_{238}t_{1}}-e^{\lambda_{238}t_{i}})\times ({\;}^{206}\mathrm{Pb}{/}^{204}{\mathrm{Pb}}_{i}\;-{\;}^{206}\mathrm{Pb}{/}^{204}{\mathrm{Pb}}_{1})\;+{\;}^{208}\mathrm{Pb}{/}^{204}{\mathrm{Pb}}_{1}$. Here $\lambda_{232}$ and $\lambda_{238}$ are the ^232^Th and ^238^U decay constants; *t*_1_ = time at which the source formed (defined by model differentiation age determined by [34]); *t_i_* = age of the sample; ${\;}^{20x}\mathrm{Pb}{/}^{204}{\mathrm{Pb}}_{i}$ = initial Pb isotope composition of sample; ${\;}^{20x}\mathrm{Pb}{/}^{204}{\mathrm{Pb}}_{1}=\mathrm{Pb}$ isotope composition of source at *t*_1_. The trend is defined by all of the points plotted, including the 14078 and 68415 data together with compositions from: 3.1–3.9 Ga Apollo 11, 12, 14, 15 and 17 basalts [52]; 2.0 Ga Chang'e-5 basalt [35]; 4.3 Ga basaltic clasts in the lunar meteorite MIL 13317 [79]; and the 3.9 Ga Apollo 16 impact melt breccia 66095 [78].

The Initial Pb isotope composition determined for 14078 indicates that a portion of the target rocks melted were derived from a source with a very high µ-value, likely in excess of 2000. The simplest case is to assume that the Pb isotope composition measured in the sample represents a single target lithology that was re-melted by an impact at 3848 ± 4 Ma, having been derived from a lunar source, the evolution of which is modelled in a single stage, starting from Canyon Diablo Troilite compositions (CDT: ^206^Pb/^204^Pb = 9.307; ^207^Pb/^204^Pb = 10.294; ^204^Pb/^206^Pb = 0.1074; ^207^Pb/^206^Pb = 1.106 [74]) at the start of the Solar System (4567 Ma [75]). In this case, the source of that lithology would have had a µ-value of approximately 1800. Using the multiple stage model of [34] for lunar Pb isotope evolution, with the source of the target lithology forming from a more evolved bulk silicate Moon composition (^206^Pb/^204^Pb = 27.932; ^207^Pb/^204^Pb = 44.356; ^204^Pb/^206^Pb = 0.0358; ^207^Pb/^206^Pb = 1.588) towards the end of lunar magma ocean crystallization (4376 ± 18 Ma), a higher source µ-value of 2400 ± 550 is calculated for the Pb in 14078 (figure 4*a*). When compared with initial compositions obtained for a range of lunar basalts and one impact melt breccia, the ^208^Pb/^204^Pb composition of 14078 is consistent with a source evolving from 4376 Ma with a ^232^Th/^238^U (κ-value) of 3.81 ± 0.13 (figure 5). In reality, the initial Pb isotope composition determined for 14078 likely represents a mixture of target lithologies that were melted during the impact event in which it was formed, and potentially some component of the impactor material. High µ-values have also been predicted for the source of the high-Al Apollo 14 basalts (e.g. 14072 and 14053 [34,52]), as well as high- and very high-K (VHK) basalts, and granitic lithologies identified as clasts in various Apollo 14 breccias [76,77]. As with 14078, the majority of these lithologies can be explained by evolution of a source with a µ-value of around 2400 (figure 4*b*), or the contribution of a highly evolved source component, potentially KREEP (µ approximately 3700 based on analyses of 15386 [34]).

Given the similarity in initial Pb isotope composition between that determined in 14078 and these other Apollo 14 samples (figure 4*b*), it appears that the effect of any contribution from impactor material was minimal. Furthermore, all of the points excluded from the sample isochron lie within the mixing triangle described earlier (§2.3) defined by the lunar Pb components (initial and *in situ* radiogenic Pb) and modern terrestrial Pb composition, as modelled by [80]. The impactor would have likely originated from a lower µ source than the Moon, and therefore had a less radiogenic Pb isotope composition than most lunar rocks (especially a KREEP-rich composition as in 14078). As such, the Pb isotope signature of the impactor would also fall within the mixing triangle, towards terrestrial compositions, and be indistinct from that of terrestrial contamination. It is, therefore, impossible to unambiguously distinguish any component of impactor material.

Analyses of Rb–Sr isotope systematics in 14078 and other Apollo 14 KREEP basalts indicate that these likely derived from sources with ^87^Rb/^86^Sr approximately 0.15–0.21 [24,61,69], similar to those predicted for the Apollo 15 KREEP basalt 15386 (^87^Rb/^86^Sr approx. 0.20 based on analyses of 15386 from [81]). The variation observed between the individual Apollo 14 KREEP basalt initial Sr isotope compositions suggests either multiple sources or a single heterogeneous source [61], which is also consistent with a slight difference in the weighted average ages of the different samples (§4.1). Nevertheless, the variation between the Apollo 14 KREEP basalts is relatively minor when compared with other Apollo 14 lithologies. For example, analyses of the high-Al basalts 14053 and 14072 [33,37] and several VHK basalts [82–85] indicate that these rocks were derived from source(s) with lower ^87^Rb/^86^Sr ratios of approximately 0.03–0.10.

Measurements of Sm–Nd isotope compositions for 14078 are lacking, but analyses of 14150 [61] indicate derivation from a source lithology with a ^147^Sm/^144^Nd ratio of approximately 0.16, similar to that of KREEP (e.g. 15386 source approximately 0.16, based on data from [86]). Equivalent Nd isotope compositions are not available for the Apollo 14 high-Al basalts, but analyses of VHK and high-K aluminous Apollo 14 basalts indicate derivation from more chondritic-like sources [84].

**Figure 6.**
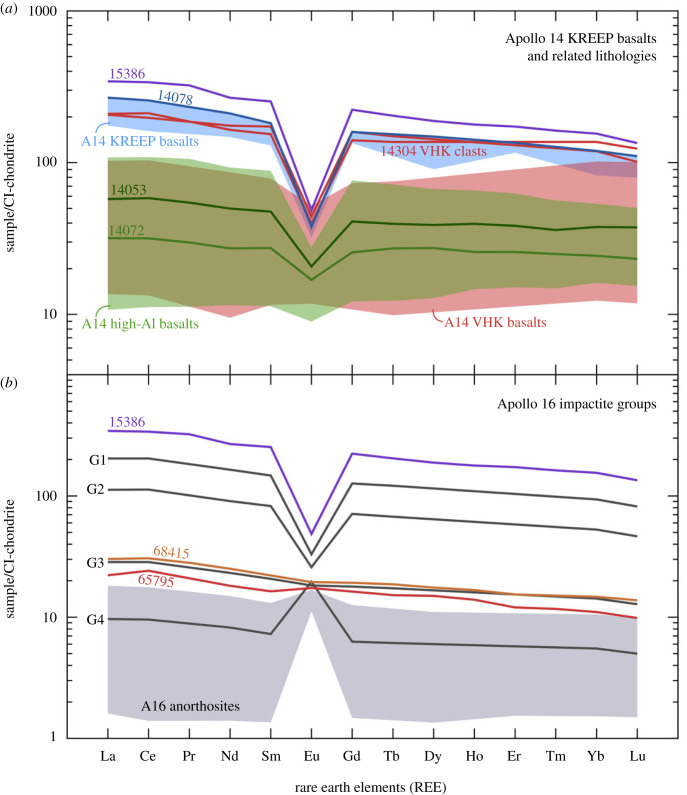
Chondrite-normalized [87] REE plots of (*a*) 14078 and related Apollo 14 lithologies [61,82,88] and (*b*) 68415 and other Apollo 16 impactite lithologies [10,30,89]. In both cases, these values are compared with the Apollo 15 KREEP basalt 15386 [90]. The REE concentrations of 14078 and the other Apollo 14 KREEP basalts indicate larger contributions of KREEP than the high-Al and VHK basalts, with the exception of two VHK basalt clasts in 14304 [88]. The REE concentrations of 68415 and other group 3 Apollo 16 impact melts indicate intermediate compositions between Apollo 16 anorthositic lithologies [91] and KREEP. The group 1 and 2 rock impact melt breccias are more KREEP-rich than the group 3 impact melts, while the group 4 impact melt breccias are more anorthositic.

Therefore, while the Pb isotope compositions of the Apollo 14 KREEP basalts (including 14078) may indicate derivation from melting of a similar target lithology to the various high-µ lithologies at the Apollo 14 landing site (e.g. high-Al and VHK basalts), the Sr and Nd isotope compositions imply that the target lithology was even more closely related to KREEP. A similar conclusion is drawn from comparison of the REE concentrations in 14078 and the other Apollo 14 KREEP basalts, which (at around 100–300 times chondritic abundances) are closer to those of Apollo 15 KREEP basalts than those from the Apollo 14 high-Al basalts (figure 6*a*) [90]. Equally, while two VHK basalt clasts from 14304 have similar REE concentrations to the Apollo 14 KREEP basalts, the majority have REE concentrations less than 100 times chondritic abundances [82,88].

#### 4.2.2. 68415

Given the lack of Pb isotope data for plagioclase and K-feldspar in 68415, it is likely that the least radiogenic composition measured in the sample (^204^Pb/^206^Pb = 0.0032 ± 0.0003 and ^207^Pb/^206^Pb = 1.15 ± 0.01) significantly underestimates the initial Pb isotope composition of the rock. Further indication of this underestimation can be seen by finding the intersection between different model paleoisochrons and the sample isochron. Starting with a single stage model, a paleoisochron originating from CDT compositions between 4567 and 3834 Ma intersects the 68415 sample isochron at ^204^Pb/^206^Pb = 0.0057 and ^207^Pb/^206^Pb = 1.51 (figure 2*f*). As with the 14078 sample, if we are first to assume that this predicted initial Pb composition resulted from the evolution of a single source, then this would equate to a source µ-value of approximately 770. A paleoisochron constructed from the differentiation point (4376–3834 Ma) of the model for lunar Pb evolution by [34] would intersect the 68415 sample isochron at ^204^Pb/^206^Pb = 0.0053 and ^207^Pb/^206^Pb = 1.43 (figure 2*f*). This would indicate evolution in a source with a slightly higher µ-value of approximately 1000 ± 25. As with 14078, there is no obvious indication in the 68415 Pb isotope compositions of Pb derived from the impactor that generated the melt, with all measured compositions being explicable by a mixing of lunar Pb and small amounts of terrestrial contamination (figure 4*a*). As with 14078, the ^208^Pb/^204^Pb composition of 68415 is consistent with a source evolving from 4376 Ma with a ^232^Th/^238^U (κ-value) of 3.81 ± 0.13 (figure 5).

These µ-values are higher than might be expected for anorthositic highland lithologies present at Apollo 16. For example, [92] estimated a µ-value of approximately 35 for anorthositic crust, based on analyses of sample 60025. But higher source µ-values (potentially greater than 1000) have been inferred before, based on analyses of other Apollo 16 samples (e.g. 60017, 62237 and 66095 [92]). More recent SIMS analyses of plagioclase in 66095 yielded an initial Pb composition indicative of evolution in a source with a µ-value of approximately 900–1000 [78]. In that case, the Pb–Pb isochron age of the sample (3909 ± 17 Ma) was consistent with it being formed during formation of the Imbrium impact basin. The Pb isotopic composition was interpreted as reflecting a mixture of components from anorthositic crustal target rocks, and a more KREEPy Pb signature potentially introduced as a result of fumarolic activity resulting from degassing of Imbrium impact ejecta [40,78,93,94]. Assuming 68415 was formed relatively locally to the Apollo 16 landing site, it is reasonable to expect that similar Pb components were incorporated into this rock as well. A slightly higher proportion of the KREEPy Pb component appears to have been incorporated into 68415, as a Pb growth curve between 3909 and 3834 Ma, starting from the 66095 initial Pb composition with a µ-value of 1000, fails to intercept the 68415 isochron (figure 4*c*).

There are no published Sm–Nd isotope compositions for either 68415 or 68416, but [16] studied the similar sample, 67559. Their results did not yield an isochron, which they interpreted to be due to a lack of Nd isotopic equilibration associated with relict plagioclase grains in the sample. However, the whole rock measurements of 67559 indicate derivation from a source with a ^147^Sm/^144^Nd of approximately 0.15, which is similar to, although even slightly lower than, than those predicted for KREEP [86].

In contrast to the Pb and Nd isotope systematics, the 68415 and 66095 samples have Sr isotope compositions indicating derivation from sources with ^87^Rb/^86^Sr approximately 0.02 (68415 [25]) and approximately 0.01 (66095 [95]). Similarly low source values of approximately 0.02–0.04 are predicted for the impact melt samples 65795 and 67559 [10,16]. This is much lower than those predicted for the sources of KREEP-rich lithologies such as Apollo 15 KREEP basalt [81], or even the group 1 [30] Apollo 16 poikilitic impact melt breccias, such as 62235 (^87^Rb/^86^Sr approx. 0.20; based on analyses from [3,95]) and 65015 (^87^Rb/^86^Sr approx. 0.16; based on analyses from [96]).

Given the high proportion of plagioclase in all of these samples (approx. 80% in 68415, 68416 and 67559) and the compatibility of Sr in plagioclase [97–99] compared to Pb (D^plag−melt^ values typically an order of magnitude lower than Sr) or Nd (D^plag−melt^ values typically several orders of magnitude lower than Sr), the Sr isotope signature in these rocks would have been less affected by the introduction of a KREEP-rich component. If the fumarolic model for introducing KREEP-rich components into the anorthositic Apollo 16 rocks and soils is correct, then the more volatile nature of Pb compared with Sr would also result in a more profound effect in the Pb isotope compositions. Furthermore, enrichments of trace elements (including Pb, Zn and Cu) in 66095 and Apollo 16 soils are correlated with enrichments in Cl, indicating the likely importance of chloride species in the mobilization of these elements [94], and Sr would not form such species. Taken together, the Sr isotope signatures in these Apollo 16 samples are likely more representative of the lunar feldspathic highland lithologies, while the Pb and Nd isotope signatures reflect the introduction of a more KREEP-rich component. Analyses of REE concentrations in these Apollo 16 samples are also consistent with small to moderate contributions of KREEP-rich material added to an anorthositic lithology (figure 6*b*).

#### 4.2.3. Bulk-rock Pb isotope compositions and ^238^U/^204^Pb ratios

Following the method of [100], it was possible to combine the isochrons for both 14078 and 68415 with previously determined bulk rock data to obtain terrestrial contamination corrected bulk ^238^U/^204^Pb ratios (µ-values). In brief, this method uses the bulk rock data and the composition of modern terrestrial Pb, and projects a line through these values to intercept the sample isochron. This intercept provides terrestrial contamination corrected bulk ^207^Pb/^206^Pb and ^204^Pb/^206^Pb ratios. A line is then constructed through the bulk rock ^238^U/^206^Pb versus ^207^Pb/^206^Pb and modern terrestrial Pb to find where it crosses the contamination corrected ^207^Pb/^206^Pb ratio, thus obtaining the corrected ^238^U/^206^Pb composition. This in turn can be combined with the corrected ^204^Pb/^206^Pb composition to obtain the bulk rock µ-value (for a more detailed description of this method and schematic diagrams, readers are referred to [100]).

In the case of 14078, there were no studies providing bulk rock U and Pb isotope data. Instead, analyses of similar samples, 14073 and 14310, were taken from [101]. The resulting bulk rock µ-values were 4490 ± 250 (using the 14073 data) and 4240 ± 220 (using the 14310 data). Bulk rock data from [73] were used to calculate the bulk µ-value of 68415, resulting in a value of 1220 ± 60. For both samples, these bulk rock µ-values are higher than the estimated µ-values of their sources (14078 source µ = 2400 ± 550; 68415 source µ = 1000 ± 25). This is most likely a result of the loss of Pb by degassing during formation of the rocks.

### 4.3. Geologic interpretation of sample ages and compositions

The similarity in the ages determined in this study for 14078 and 68415 is interpreted to be coincidental. In each case the initial Pb isotope compositions (measured for 14078 and inferred from model calculations for 68415), as well as previously determined Sr and Nd isotope compositions, and REE concentrations, are distinct from each other, but consistent with observations of other samples and lithologies from the same landing sites. While it might theoretically be possible for an impact event to generate impact melt exhibiting such heterogeneities, there is a lack of obvious candidates for sufficiently large post-Imbrium basins that could have generated these specific ranges of melt compositions and deposited material near the Apollo 14 and 16 landing sites. An early study of the Apollo 16 landing site proposed that 68415 and other Cayley plains samples might have been generated by the formation of the Orientale basin [102]. While 68415 and the other group 3 Apollo 16 impact melt samples may be a reasonable compositional match for recent estimates of Orientale impact melt (in particular FeO and TiO_2_ concentrations based on Clementine and Lunar Prospector data [103–105]), 14078 and the Apollo 14 KREEP basalts have significantly higher FeO and TiO_2_ concentrations [24]. As such, the simplest explanation is that these samples represent melt generated in separate impacts.

In order to generate the two groups of Apollo 14 KREEP basalts (including 14078), the target lithologies would have been similar to the high-Al and VHK basalts in the area but incorporating a more significant KREEP component. In particular, the two high-REE 14304 VHK basalt clasts analysed by [88] would appear to have similar trace element concentrations to the KREEP basalts, despite lacking isotopic measurements for additional corroboration.

In the case of 68415 and the relation to other Apollo 16 impactites, similarities and distinctions in the sample ages are broadly consistent with previous compositional and petrologic classifications [10,16,23,29,30]. The Imbrium basin-forming impact resulted in the emplacement of the Descartes and Cayley formations [89,106] and the generation of the majority of group 1 and 2 Apollo 16 impact melt breccias. A weighted average of the similarly aged ANMR/group 3 samples indicates they were formed at 3831 ± 8 Ma (MSWD = 1.3; *p* = 0.20). Despite the earlier suggestion of the Orientale basin as a source for these rocks, the trace element and isotopic compositions of the samples are more indicative of an impact local to the Apollo 16 site, resulting in a mix of anorthositic highland rocks with the more trace-element-enriched Imbrium ejecta. This interpretation would also be consistent with the older ^40^Ar/^39^Ar age from 68416 [26], interpreted as resulting from relict material in the impact melt, being similar to that determined for the formation of the Imbrium impact basin.

While the Imbrium basin may have been the last very large (greater than 1000 km diameter) lunar impact basin to form, the ages of the Apollo 14 KREEP basalts and the post-Imbrium aged Apollo 16 breccias provide evidence for relatively intense impact bombardment of the Moon continuing after the Imbrium event. This interpretation is further supported by the conclusion that neither of the samples studied here likely represent ejecta from the Orientale basin, which must therefore represent a separate post-Imbrium impact event. As such, even with the Imbrium basin forming at 3922 ± 12 Ma [17], it is still reasonable to assume that heavy bombardment in the inner Solar System would have continued to have had a significant effect on the earliest stages of terrestrial crustal evolution. That said, it should be noted that evidence for a handful of individual impact events on one planetary body is not, in itself, sufficient to confirm the occurrence of a broader LHB throughout the inner Solar System. This is a task for future reviews to collate and statistically assess new evidence from all available impactite dating studies in much the same way as has been done previously (e.g. [7,9]).

## 5. Conclusion

A Pb–Pb isochron age of 3848 ± 4 Ma was determined for the Apollo 14 KREEP basalt 14078. A weighted average of this and previously determined Apollo 14 KREEP basalt ages indicates that they were formed by impacts at 3849 ± 5 Ma and 3876 ± 17 Ma. An initial Pb isotopic composition determined from the 14078 measurements indicates that the sample was derived from target lithologies with high-µ (2400 ± 550) sources, consistent with the predicted sources of other Apollo 14 lithologies (e.g. high-Al and VHK basalts), although it is noted that Sr and Nd isotope compositions and REE concentrations of the high-Al and VHK basalts indicate a lower proportion of KREEP.

A similar Pb–Pb isochron age of 3834 ± 11 Ma was determined for the Apollo 16 impact melt sample 68415. The majority of other ANMR/group 3 Apollo 16 impact melt rocks have consistent ages, resulting in a weighted average age of 3831 ± 8 Ma. While it was not possible to directly determine an initial Pb composition from the 68415 measurements in this study, modelled initial Pb compositions indicate the sample was derived from lithologies with higher source µ-values (1000 ± 25) than might typically be expected of anorthositic rocks, suggesting a minor contribution of KREEP. When combined with previous analyses of Apollo 16 impactite lithologies, it is concluded that 68415 and the other ANMR/group 3 samples were formed by an impact local to the Apollo 16 landing site, resulting in the mixture of anorthositic highland lithologies with more KREEP-rich Imbrium ejecta.

## Data Availability

All data are included in the supplementary figures and tables [107].
